# Objective characterization of displacement of the frontal aslant tract in low-grade glioma: a quantitative tractography study

**DOI:** 10.1007/s00234-026-03968-z

**Published:** 2026-04-01

**Authors:** A. L. Mahmoodi, L. Smolders, M. J. F. Landers, G. J. M. Rutten, H. B. Brouwers

**Affiliations:** 1 Department of Neurosurgery, Elisabeth-TweeSteden Ziekenhuis, Tilburg, Netherlands; 2 Department of Mathematics and Computer Science, Eindhoven University of Technology, Eindhoven, Netherlands

**Keywords:** Tractography, White matter tract alteration pattern, Spatial metrics, Dice similarity coefficient, Hausdorff distance, Glioma

## Abstract

**Purpose:**

We investigated whether quantitative tractography-based spatial metrics can objectively characterize visual tumor-induced white matter (WM) tract displacement in low-grade glioma. Specifically, the frontal aslant tract (FAT) was evaluated using the weighted Dice similarity coefficient (wDSC) and Hausdorff distance (HD) as spatial metrics.

**Methods:**

We retrospectively analyzed 45 patients with IDH-mutated frontal low-grade glioma (LGG), in whom preoperative diffusion-weighted MRI and probabilistic tractography of the FAT were performed. Patients were classified into one of three spatial WM tract alteration pattern groups (displacement, infiltration, combination) based on expert visual assessment. The wDSC and HD were investigated in both MNI-152 normalized and native anatomical space. In normalized space, inter-hemispheric comparisons (within patients) and intra-hemispheric comparisons (across patients) of spatial metrics were performed. In native space, comparisons were performed within patients, between mirrored ipsilesional and contralesional tracts.

**Results:**

In normalized MNI space, FAT displacement led to a decreased wDSC and increased HD during inter-hemispheric comparisons within patients, while infiltrated tracts showed no such differences. Conversely, intra-hemispheric spatial metrics, assessed across patients and independently of contralateral tract anatomy, could not differentiate FAT displacement from infiltration in MNI space. In native space, displacement of the FAT could be characterized objectively, using tract mirroring.

**Conclusion:**

Visual tumor-induced displacement of the FAT can be objectively confirmed with measurable geometric tract alterations using spatial metrics. While displacement could be characterized through inter-hemispheric comparisons in MNI space, native space might be more robust for detecting intra-hemispheric geometric differences. This study provides the foundation for an objective, quantitative framework to evaluate spatial WM tract alterations in glioma. These results might advance non-invasive radiomics-based tumor subtype predictions.

**Supplementary Information:**

The online version contains supplementary material available at 10.1007/s00234-026-03968-z.

## Introduction

Magnetic resonance (MR) tractography is a non-invasive 3D neuroimaging technique used to visualize white matter (WM) tracts in the brain. It is increasingly used as a clinical tool for glioma surgery planning, as it can aid in preoperative and intraoperative decision making [1–3]. In glioma surgery, a complex onco-functional balance exists between safely maximizing the extent of resection (EOR) and preserving neurological function [4–7]. Although a larger EOR is related to prolonged survival rates, it also increases the risk of postoperative functional deficits [8–10]. Optimizing this balance is particularly challenging in low-grade gliomas (LGG), which can infiltrate and spread diffusely along WM tracts, often leading to significant anatomical alterations of these tracts [11, 12]. Several distinct patterns of glioma-induced spatial WM tract alterations have been described: WM tract displacement, infiltration, destruction, or vasogenic edema surrounding tracts [13–16]. Recent literature has highlighted that identifying these patterns relies heavily on subjective visual assessments of tractography output, i.e. tractograms, as no objective and user-independent quantitative classification system currently exists [17, 18]. Developing such as system would not only enhance our understanding of glioma growth behavior, but also improve the comparability of tractography studies. Therefore, objective classifications of the spatial interactions between LGG and surrounding WM tracts could ultimately help to further optimize the onco-functional balance in glioma surgery.

Over the years, spatial metrics have been introduced to the field of tractography, mainly to validate the accuracy and precision of tractography frameworks [19]. Most of these metrics can be broadly categorized into overlap- or distance-based metrics. A commonly used overlap-based metric is the Dice-Sørensen coefficient (referred to as the Dice similarity coefficient [DSC]), which quantifies the 3D overlap between two image segmentations [20–22]. A frequently used distance-based metric is the Hausdorff distance (HD), which measures the maximum distance between two sets of points [20, 23]. Together, the DSC and HD describe both the overall discrepancy (DSC) and the regional discrepancy (HD) between two image segmentations. The unique properties of these metrics might also facilitate the objective characterization of spatial WM tract displacement, using quantitative tractography.

In the current study, we investigate whether the DSC and HD can quantitatively characterize visually assessed WM tract displacement in LGG. We hypothesize that displacement results in measurable geometric tract deviations, that are detected by the spatial metrics. Furthermore, we hypothesize that infiltration does not alter spatial metrics, as it preserves tract geometry within physiological limits and the metrics are designed to exclusively characterize displacement.

## Methods

### Study population

**Fig. 1:**
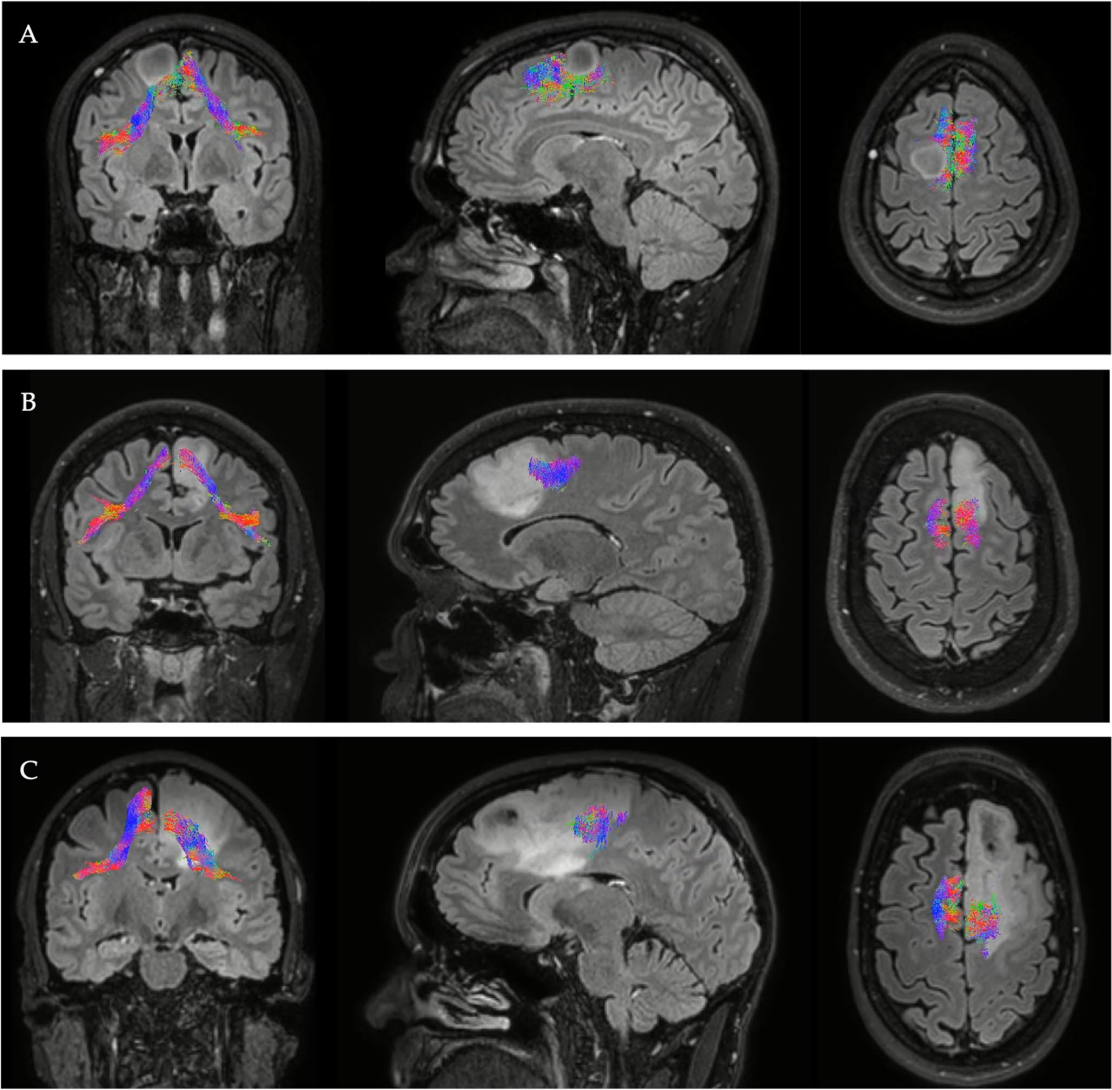
Illustrative cases of visually assessed white matter tract alteration patterns. **A**) Displacement of the FAT. **B**) Infiltration of the FAT. **C**) Combined displacement and infiltration of the FAT. Streamlines are visualized as color-coded direction maps: red = left-right, blue = superior-inferior, green = anterior-posterior

We retrospectively analyzed a cohort of patients with histopathologically confirmed IDH-mutated LGG, in whom preoperative diffusion-weighted (DW) MRI scans were obtained at the Elisabeth-TweeSteden Hospital in Tilburg, the Netherlands. Inclusion occurred between April 2011 and July 2021. This study focused on the frontal aslant tract (FAT). The FAT connects the inferior frontal gyrus and the (pre-)supplementary motor area (SMA), and is associated with speech, language and executive functions [24]. Inclusion criteria were (1) a tumor located in the frontal lobe; (2) a shortest distance between the tumor and the left or right FAT of less than two centimeters at any point and (3) the left or right FAT being visually displaced, infiltrated or both by the tumor. Visual assessments of our cohort were previously conducted as described in Landers et al. [25] by four blinded experts: two neuroradiologists, one neurosurgeon specialized in neuro-oncology, and one neurosurgical resident, all with extensive clinical tractography experience. Assessment differences were re-evaluated after which consensus was reached for all cases. This resulted in a ground truth classification, consisting of three groups of distinct spatial WM tract alteration patterns: (1) displacement of the FAT, (2) infiltration of the FAT, (3) combined displacement and infiltration of the FAT (Fig. 1). Patients were excluded if tractography output quality was insufficient to calculate spatial metrics.

### Diffusion-weighted imaging & tractography

DW-MRI was acquired on a Philips Achieva 3T MRI scanner (50 b = 1500 volumes, 6 b = 0 volumes, 2 mm isotropic voxel size). Probabilistic tractography of the bilateral FAT was performed, using an automated subject-specific pipeline, as previously described [26]. This tractography pipeline performs constrained spherical deconvolution (CSD) using functions from the open source MRtrix software [27]. Tractography was seeded in the SMA and pre-SMA regions, with target regions being the pars triangularis and the pars opercularis. Tractography was performed with the iFOD2 algorithm as implemented in MRtrix3, using the following parameters: step size 0.2 mm, radius of curvature threshold 1 mm, fiber orientation distribution (FOD) amplitude threshold 0.09, maximum number of ten thousand attempts for each seed voxel and a target number of 5000 streamlines in total. See Meesters et al. [26] for the complete detailed tractography configuration.

### Streamline processing

**Fig. 2:**
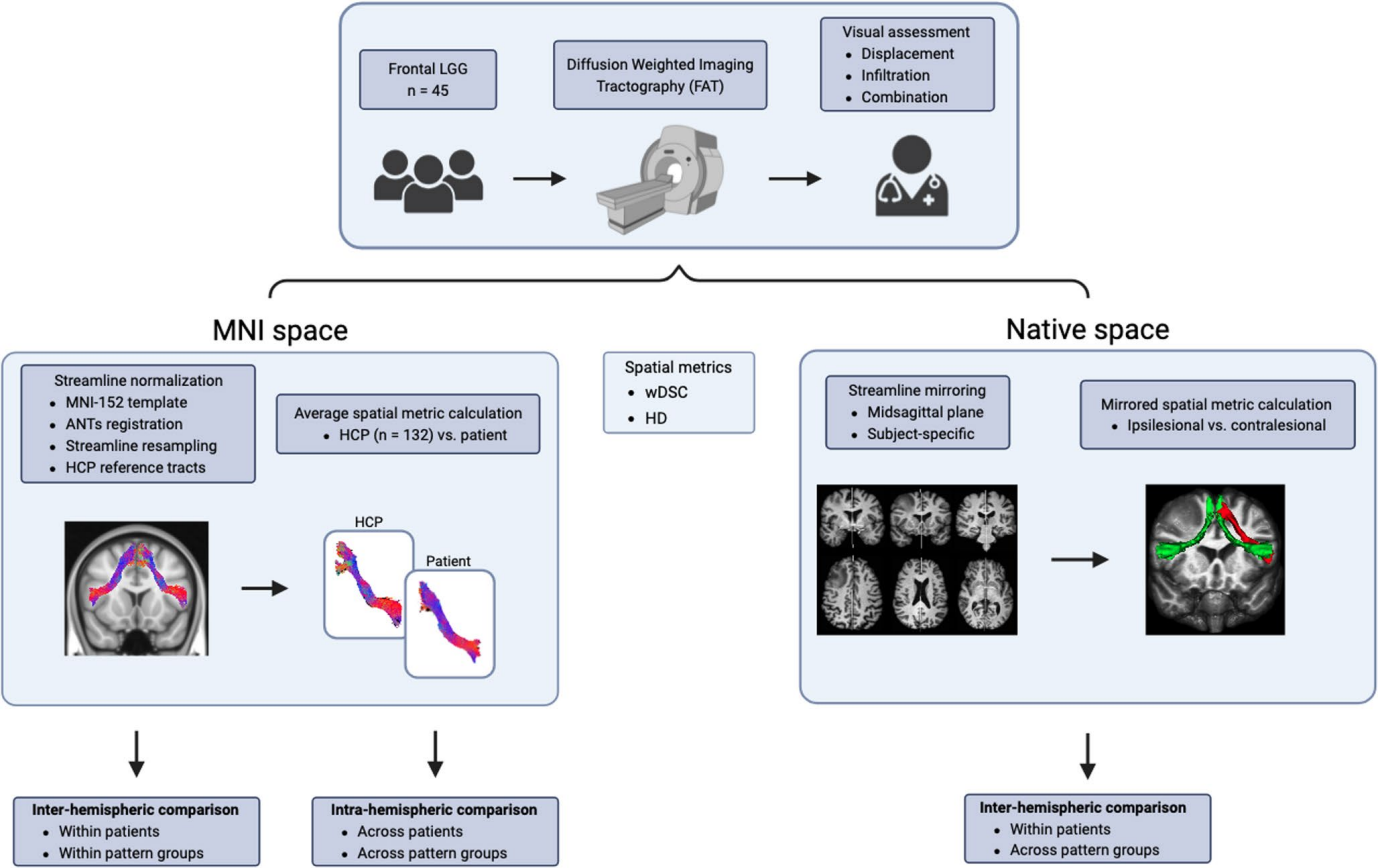
Data flow and methodological steps. Study population, diffusion-weighted imaging, tractography and visual WM tract alteration pattern assessment (top block). MNI space streamline processing and spatial metrics calculation (left block). Native space streamline processing and spatial metrics calculation (right block). Inter- and intra-hemispheric comparisons (bottom blocks)

Streamlines were processed differently for analyses in normalized anatomical space and patient-specific native space. Normalized space enables intra-hemispheric comparisons across subjects by aligning tractograms to a standardized anatomical template. Native space facilitates inter-hemispheric analyses within subjects, preserving subject-specific anatomical characteristics. Figure 2 illustrates the data flow and methodological steps.

#### MNI space

To enable comparisons of spatial metrics across subjects, streamlines of the FAT were transformed to the normalized Montreal Neurological Institute (MNI) space [28]. In brief, the T1-weighted MRI scan of each patient was registered to the MNI-152 template using Advanced Normalization Tools (ANTs) software [29]. Next, the resulting non-linear transformations were applied to the FAT tractograms. Subsequently, lengthwise normalization of streamlines was performed by resampling streamlines to 500 equally distant data points, to account for variations in streamline length both between and within patients. Supplementary Fig. 1 shows the normalized patient streamlines as a probabilistic heatmap for each WM tract alteration pattern. To account for normal physiological variations in the course and location of the FAT after MNI transformation, DWI data of 132 healthy subjects from the Human Connectome Project (HCP) were processed similarly to patient data [30]. Subsequently, the spatial metrics (see section below) were calculated between a patient and each of the 132 HCP subjects, after which an average metric was obtained for each patient. This way, alterations in patient FAT anatomy were quantified as deviations from healthy physiological variation.

#### Native space

In native space, the spatial metrics (see section below) were calculated between the contralesional and mirrored ipsilesional FAT streamlines, without involvement of HCP subjects, as the native space inherently accounts for each subject’s inter-hemispherical anatomical variations. Streamlines were mirrored from the ipsilesional hemisphere onto the contralesional hemisphere, using subject-specific midsagittal planes (MSP) that were generated for T1-weighted MRI scans as described by Hu et al. [31].

### Spatial metrics

#### Weighted Dice similarity coefficient

**Fig. 3:**
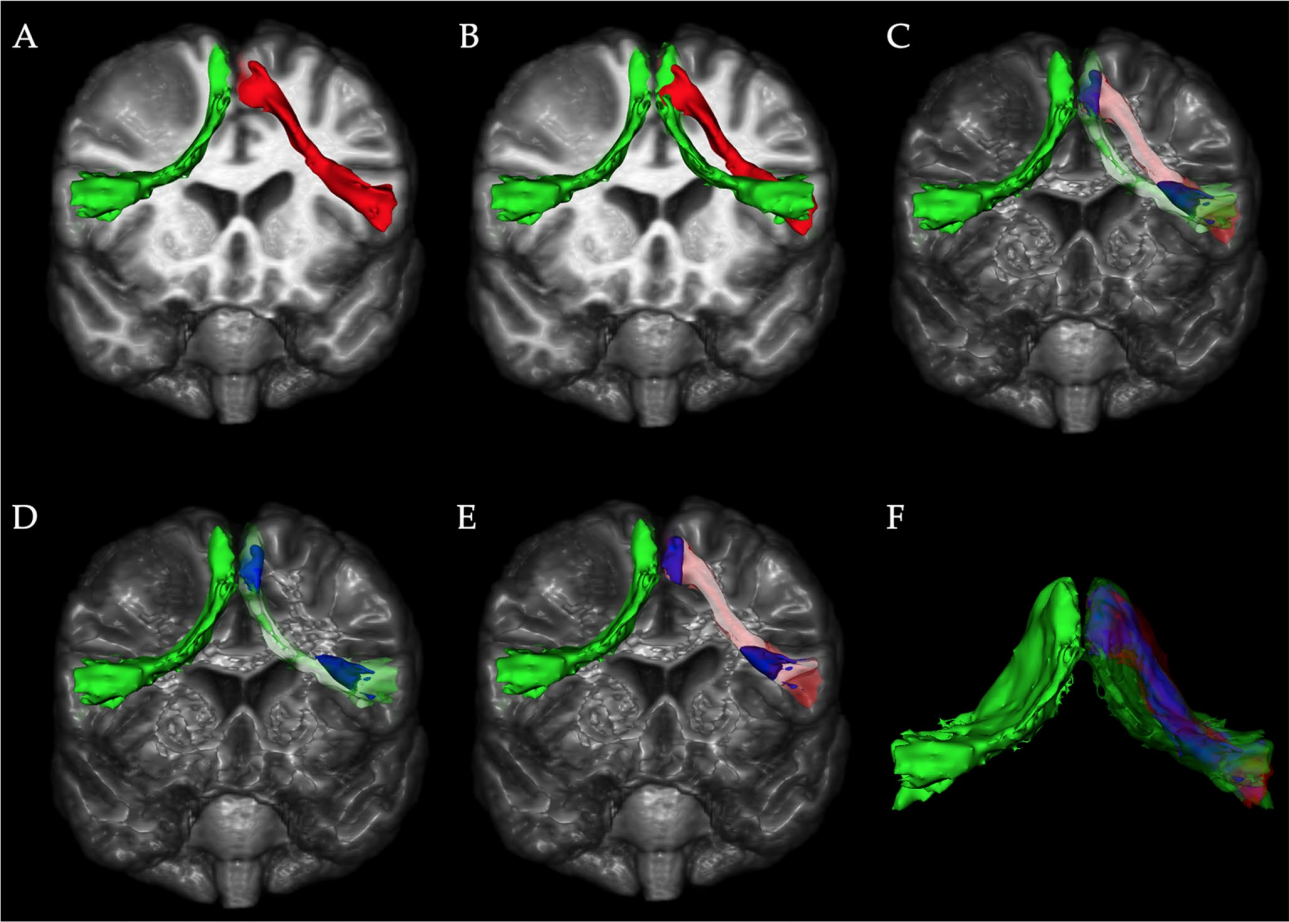
Visualization of the weighted Dice similarity coefficient (wDSC) using 3D modeling in a patient with visual tumor-induced displacement of the right FAT. A relatively low wDSC of 0.53 was calculated for this case. **A**) Right FAT (green) is visibly displaced by tumor, compared to contralesional left FAT (red). **B**) Right FAT is mirrored across the subject-specific midsagittal plane. **C**) Overlapping regions (blue) between left FAT (red) and mirrored right FAT (green) are determined. **D)/E**) Overlapping voxels are isolated from each individual tractogram to calculate wDSC. **F**) Visualization of closed surface representations of entire fiber bundles independent of image slice, including overlapping parts. *Note*: figure applies to native space, for MNI space analyses, average spatial metrics are calculated independently for each hemisphere between patient and HCP subjects

The DSC quantifies 3D overlap between two tractograms. As varying numbers of streamlines can traverse each voxel, a weighted version of the DSC (wDSC) was calculated to assign greater importance to voxels containing multiple streamlines [32]. In brief, density maps were generated from FAT streamlines, representing the number of streamlines passing through each voxel. Subsequently, binary and weighted density maps were created. In binary density maps, voxels containing any number of streamlines were assigned a value of 1, whereas empty voxels were set to 0. In weighted density maps, voxel values represented the fraction of the total number of streamlines passing through each voxel. Overlap images were created by multiplying the voxel values in the respective binary density maps. The weighted values of overlapping voxels were then extracted from the weighted density maps, using the overlap image as a mask. Finally, the wDSC was calculated by dividing the sum of the weighted values of overlapping voxels by the sum of the binary voxel values of each tractogram. Figure 3 illustrates the wDSC in an example case in native space based on mirrored tracts, visualized using the 3D Slicer software [33].

#### Hausdorff distance

The HD quantifies the maximum distance between two tractograms. Calculation of the HD $h\left(A,B\right)$ between objects *A* and *B* requires a representation of these objects as sets of points, which consist of locations along the streamlines of the FAT. The FAT of each patient or HCP subject was reduced to a single streamline that represented the subject-specific 3D center of the tract. These core streamlines were obtained by calculating average (x, y, z) coordinates for each of the 500 points along all FAT streamlines. To reduce the known sensitivity of the HD to outliers [20], which could be caused by the natural tendency of the FAT streamlines to fan outward near the ends of the tract, the inner 80% of the 500 points of each core streamline’s length was used to calculate the HD.

**Fig. 4:**
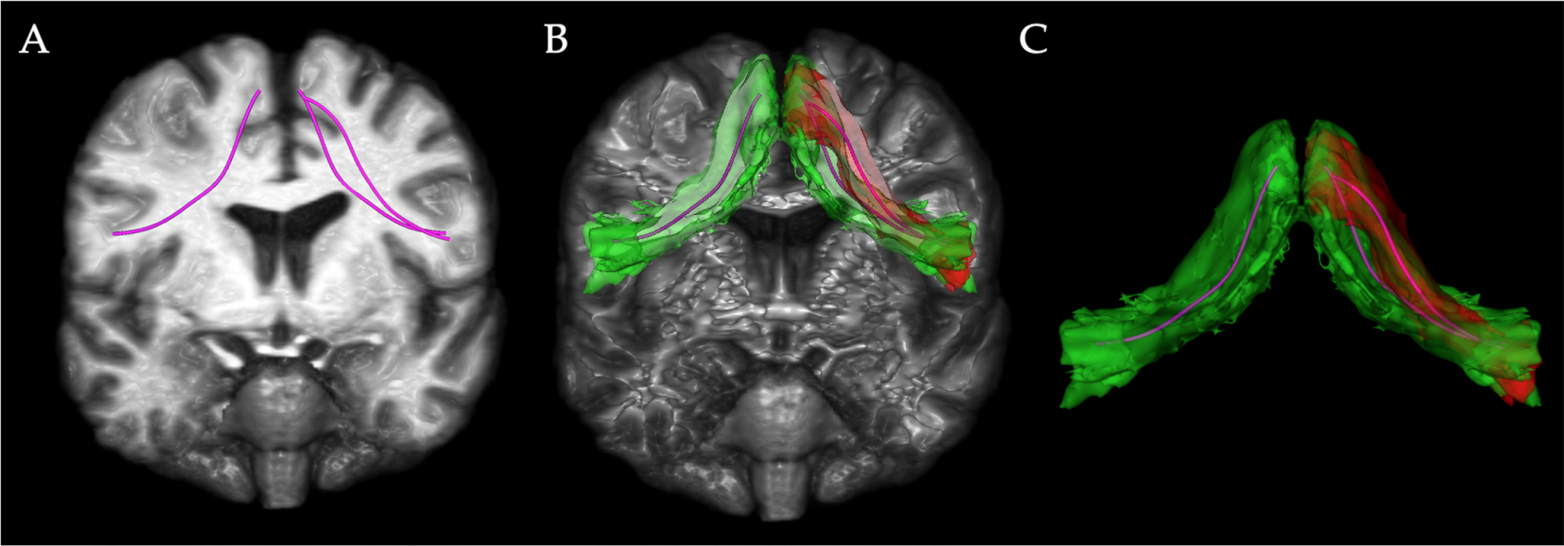
Visualization of the Hausdorff distance (HD) using 3D modeling in a patient with visual tumor-induced displacement of the right FAT. A relatively high HD of 7.49 mm was calculated for this case. **A**) Core streamlines of the right, left and mirrored right FAT. The HD is calculated within the left hemisphere as the greatest value among the shortest distances from each point of the mirrored FAT core streamline to the left FAT core streamline. **B**)**/C**) Visualization of core streamlines within closed surface representations of entire fiber bundles, independent of image slice. *Note*: figure applies to native space, for MNI space analyses, average spatial metrics are calculated independently for each hemisphere between patient and HCP subjects

It should also be noted that the classic HD is directed, meaning that $h\left(A,B\right)$ and $h\left(B,A\right)$ are not necessarily equal. We calculated the directed HD from the ipsilesional tract towards the contralesional tract (or in MNI space from ipsilesional to HCP tract). This approach ensured that the length of the ipsilesional tract acted as the limiting factor in the calculation, preventing artificially inflated HD when contralesional streamline lengths (or HCP streamlines in MNI space) extended beyond the boundaries of ipsilesional streamlines. Figure 4 illustrates the HD in an example case in native space based on mirrored tracts, visualized using the 3D Slicer software [33].

### Statistics

Statistical analyses were performed with SPSS Statistics, version 29 [34]. If data were non-normally distributed or other assumptions of statistical tests were not met, non-parametric alternatives were applied. Patient characteristics including age, sex, affected hemisphere and tumor volume were compared between study groups. Numerical variables were analyzed using one-way ANOVA, followed by Tukey’s post-hoc test. Categorical variables were analyzed using Fisher’s exact tests. Comparisons of spatial metrics between the three WM tract pattern groups were performed using Kruskal-Wallis tests and Dunn’s post-hoc tests. Comparisons of spatial metrics between hemispheres within individual WM tract pattern groups were performed using Wilcoxon signed-rank tests. Statistical significance was defined as a p-value of < 0.05.

## Results

### Descriptive statistics

During the study period, 64 patients underwent DW-MRI followed by MR tractography, of whom 45 met the inclusion criteria. Twelve patients were excluded based on the minimal tumor-tract distance, four patients based on tumor location, three patients due to insufficient tractography output quality. Patient characteristics are summarized in Table 1. Mean tumor volume showed a significant difference between the three study groups (*p* = .045), with larger mean volumes observed in the combination WM tract pattern group compared to both the displacement group (*p* = .048) and infiltration group (*p* = .047). No significant differences were found for the remaining patient characteristics (age, sex, affected hemisphere). Tractography of the FAT was also performed in 132 HCP reference subjects, using the same pipeline as for patient data. Table 2 presents the variability in the wDSC and the HD in the healthy reference HCP population. Mean wDSC varied from 0.75 (native space) to 0.78 (MNI space). Mean HD varied from 4.54 (native space) to 5.50 (MNI space).

**Table 1:** Patient characteristics

		Total (*n* = 45)	WM tract alteration pattern			
			Displacement (*n* = 23)	Infiltration (*n* = 10)	Combination (*n* = 12)	*p*
Mean age (SD, range)		41.4 (11.8, 21–66)	40.1 (10.5, 21–59)	41.5 (11.6, 24–60)	43.8 (14.7, 23–66)	0.686
Sex (*n*, %)	Male	29 (64%)	14 (61%)	8 (80%)	7 (58%)	0.556
	Female	16 (36%)	9 (39%)	2 (20%)	5 (42%)	
Affected hemisphere (*n*, %)	Left	25 (56%)	10 (43%)	6 (60%)	9 (75%)	0.241
	Right	20 (44%)	13 (57%)	4 (40%)	3 (25%)	
Mean tumor volume in cm^3^ (SD, range)		46.4 (45.6, 3.0-189.6)	33.2 (31.7, 3.0-141.4)	32.4 (23.6, 3.5–74.6)	83.5 (61.9, 7.4-189.6)	0.045*

Tumor volume was evaluated using Welch’s ANOVA and the Games-Howell post hoc test as the assumption of homogeneity of variances was violated

* Mean tumor volume was significantly higher in the combination group compared to the displacement group (*p* = .048) and compared to the infiltration group (*p* = .047)

**Table 2:** Healthy reference distributions of spatial metrics in human connectome project subjects

	wDSC (proportion) – mean (SD)	Hausdorff dis. (mm) – mean (SD)
**MNI space (inter-subject)**		
Right hemisphere (*n*=132)	0.76 (0.07)	5.38 (1.03)
Left hemisphere (*n*=132)	0.78 (0.05)	5.50 (1.12)
**Native space (intra-subject)**		
Left-right mirrored (*n*=132)	0.75 (0.10)	4.54 (1.76)I

In MNI space spatial metrics were calculated separately for each hemisphere across HCP subjects. In native space tractograms were mirrored and one spatial metric was calculated within each subject

### Inter-hemispheric spatial metrics are altered in displaced tracts but not in infiltrated tracts in MNI space

Inter-hemispheric differences in wDSC and HD, i.e., within patients, were evaluated among the visually displaced or infiltrated FAT, using HCP-based average metrics to account for physiological variations after MNI transformation (Table 3).

In the visually displaced group, the wDSC was significantly lower in the ipsilesional hemisphere compared to the contralesional hemisphere (*p* = .001) Conversely, the ipsilesional HD was significantly higher compared to the contralesional hemisphere (*p* = .001). In the visually infiltrated group, no significant differences between hemispheres were found for either the wDSC (*p* = .799) or the HD (*p* = .386). In the simultaneously displaced and infiltrated group, the wDSC was significantly lower in the ipsilesional hemisphere compared to the contralesional hemisphere (*p* = .010), while the ipsilesional HD was significantly higher compared to the contralesional hemisphere (*p* = .005). These findings indicate that visual displacement of the FAT results in quantifiable geometric tract alterations, while infiltrated tracts retain their geometry.

**Table 3:** Inter-hemispheric (i.e. within patient) comparison of spatial metrics among WM tract alteration patterns in MNI space

		Ipsilesional		Contralesional	wDSC (d)		*p* (a – c)	*p* (b – d)
		Hausdorff. dis. (a)	wDSC (b)	Hausdorff. dis. (c)				
Displacement (*n* = 23)	Median 95% CI IQR	6.78 5.46–8.32 5.20–9.39	0.66 0.60–0.74 0.56–0.76	5.16 4.75–5.75 4.70–5.80	0.77 0.76–0.80 0.75–0.80	*r*_*rb*_	**0.001** 0.48	**0.001** − 0.47
Infiltration (*n* = 10)		6.35 5.34–7.86 5.33–8.00	0.69 0.62–0.74 0.61–0.74	5.62 5.02–7.34 5.07–6.64	0.68 0.62–0.76 0.61–0.77	*r*_*rb*_	0.386 0.19	0.799 0.06
Combination (*n* = 12)		11.22 8.67–13.85 8.65–14.25	0.42 0.26–0.70 0.25–0.65	5.97 5.11–9.52 5.15–10.21	0.71 0.56–0.76 0.54–0.77	*r*_*rb*_	**0.005** 0.58	**0.010** − 0.53

Analyses performed using Wilcoxon signed-rank test. 95% CI: 95% confidence interval. IQR: interquartile range

Effect sizes are displayed as rank-biserial correlation (*r*_*rb*_)

### Intra-hemispheric spatial metrics do not objectively characterize tract displacement in MNI space

Intra-hemispheric differences in wDSC and HD, i.e., across patients, were evaluated among the visually displaced or infiltrated FAT, using HCP-based average metrics to account for physiological variations after MNI transformation (Table 4).

Intra-hemispheric comparisons of the spatial metrics showed that the ipsilesional wDSC was significantly lower in the simultaneously displaced and infiltrated group compared to both the solely displaced group (*p* < .01) and the solely infiltrated group (*p* < .05) (Table 4). Conversely, the ipsilesional HD was significantly higher in the simultaneously displaced and infiltrated FAT group compared to both the solely displaced group (*p* < .01) and the solely infiltrated group (*p* < .01). However, no significant differences were observed between the displaced or infiltrated groups for either metric. These results suggest that the intra-hemispheric wDSC and HD, assessed independently of contralateral tract anatomy, cannot differentiate displacement from infiltration of the FAT in MNI space. 

**Table 4:** Intra-hemispheric (i.e. across patient) comparison of spatial metrics among WM tract alteration patterns in MNI space

		Displacement (*n*=23)	Infiltration (*n*=10)	Combination (*n*=12)	*p*		Post-hoc analysis		
							Disp-Inf	Disp-Comb	Inf-Comb
**wDSC (proportion)**									
Ipsilesional	Median	0.66	0.69	0.42	**.013**	*r*_*rb*_	n.s. – 0.01	** 0.56	* 0.49
	95% CI	0.60 – 0.74	0.62 – 0.74	0.26 – 0.70					
	IQR	0.56 – 0.76	0.61 – 0.74	0.25 – 0.65					
Contralesional		0.77	0.68	0.71	**.006**	*r*_*rb*_	* 0.50	** 0.55	n.s. – 0.04
		0.76 – 0.80	0.62 – 0.76	0.56 – 0.76					
		0.75 – 0.80	0.61 – 0.77	0.54 – 0.77					
**Hausdorff dis. (mm)**									
Ipsilesional		6.78	6.35	11.22	**.007**	*r*_*rb*_	n.s. 0.11	** – 0.55	** – 0.59
		5.46 – 8.32	5.34 – 7.86	8.67 – 13.85					
		5.20 – 9.39	5.33 – 8.00	8.65 – 14.25					
Contralesional		5.16	5.62	5.97	.068	*r*_*rb*_	n.s. – 0.24	* – 0.45	n.s. – 0.18
		4.75 – 5.75	5.02 – 7.34	5.11 – 9.52					
		4.70 – 5.80	5.07 – 6.64	5.15 – 10.21					

Analyses performed using Kruskal-Wallis test and post-hoc Dunn’s test. *95% CI* 95% confidence interval; *IQR* interquartile range

Effect sizes are displayed as rank-biserial correlation (*r*_*rb*_)

*p value < 0.05

**p value < 0.01

### Displaced tracts demonstrate increased HD and decreased wDSC compared to infiltrated tracts in native space

The spatial metrics in native space were calculated by mirroring the ipsilesional FAT to the contralesional hemisphere using subject-specific MSPs (Table 5).

In the displaced group, the mirrored wDSC was significantly lower compared to the infiltrated group (*p* < .01), while the mirrored HD was significantly higher compared to the infiltrated group (*p* < .05). In the simultaneously displaced and infiltrated group, the mirrored wDSC was significantly lower compared to the solely infiltrated group (*p* < .01) and the mirrored HD was significantly higher (*p* < .01). No significant differences were found between the solely displaced group and the simultaneously displaced and infiltrated group for either metric. These results indicate that visual displacement of the FAT is quantitatively captured by the wDSC and HD, using tract mirroring in native space.

**Table 5:** Comparison of ipsilesional – contralesional mirrored spatial metrics among WM tract alteration patterns in native space

		Displacement (*n*=23)	Infiltration (*n*=10)	Combination (*n*=12)	*p*		Post-hoc analysis		
							Disp-Inf	Disp-Comb	Inf-Comb
Mirror wDSC	Median	0.58	0.77	0.52	**.003**	*r*_*rb*_	** - 0.66	n.s. 0.07	** 0.65
	95% CI	0.50 – 0.64	0.68 – 0.79	0.30 – 0.70					
	IQR	0.45 – 0.65	0.68 – 0.79	0.31 – 0.68					
Mirror Hausdorff dis.		7.67	5.29	12.16	**.004**	*r*_*rb*_	* 0.47	n.s. - 0.31	** - 0.79
		5.94 – 9.74	3.72 – 6.64	7.48 – 16.61					
		5.92 – 10.95	3.71 – 6.48	7.76 – 16.00					

Analyses performed using Kruskal-Wallis test and post-hoc Dunn’s test. *95% CI* 95% confidence interval; *IQR* interquartile range

Effect sizes are displayed as rank-biserial correlation (*r*_*rb*_)

*p value < 0.05

**p value < 0.01

## Discussion

This study shows that visual tumor-induced displacement of the FAT can be objectively confirmed with measurable geometric tract alterations using spatial metrics. Spatial metrics were investigated in both MNI-normalized and native anatomical space, with native space yielding the most reliable results. In MNI space, FAT displacement led to a decreased wDSC and increased HD during inter-hemispheric comparisons within patients. Conversely, intra-hemispheric spatial metrics, assessed independently of contralateral tract anatomy, could not differentiate FAT displacement from infiltration in MNI space. In native space, displacement of the FAT could be characterized objectively, using tract mirroring. These findings demonstrate the ability of spatial metrics to objectively characterize visually assessed WM tract displacement in glioma.

The differences between intra- and inter-hemispheric results in MNI space may stem from how tractograms were normalized. To enable comparisons between patients, subject anatomy was aligned to the standardized MNI-152 template, instead of generating a cohort-specific template that has been described to improve registration accuracy [35, 36]. This approach allowed us to avoid introducing unwanted subject interdependencies. A cohort-specific template based on the current sample would be biased towards the anatomy of visually displaced cases, as these predominated. Additionally, the exclusive inclusion of frontal gliomas would skew this template towards frontal anatomical distortions. Taken together, a cohort-specific template could have attenuated a possible relationship between visual displacement and our spatial metrics. Nevertheless, using a standardized MNI normalization template did inherently introduce some anatomical errors, by aligning different brain anatomies to one average template. These errors may have disproportionately affected intra-hemispheric analyses, which relied on comparing spatial metrics across different patients. In contrast, native space preserved the subject-specific anatomy, possibly making it a more robust imaging space for detecting intra-hemispheric geometric differences across subjects.

No previous studies have used spatial metrics to characterize WM tract displacement in glioma. Quantifying these alterations would facilitate comparisons across studies, underscoring the relevance of the current work. Furthermore, quantifying spatial tumor-tract interactions might enhance non-invasive tumor characterization, as recent evidence suggests that LGG subtype demonstrate distinct WM tract alteration patterns. For instance, in a cohort of frontal LGG, astrocytomas predominantly displaced the FAT, whereas oligodendrogliomas showed a tendency for infiltration [25]. Together, these considerations highlight the value of spatial metrics in advancing glioma research.

A key strength of this study is the use of two established spatial metrics, the Dice similarity coefficient and the Hausdorff distance, to objectively quantify displacement of the FAT [20]. The Dice coefficient is robust against outliers from fanning streamlines near tract ends, making it an appropriate overlap-based metric to assess overall tract displacement [20]. A modified weighted version was utilized to account for varying streamline densities along tracts, further optimizing the metric’s accuracy [32]. The Hausdorff distance is a suitable distance-based metric for measurements along the complex shapes of WM tracts to assess regional tract displacement [20]. Another strength is that we highlighted some of the shortcomings of the commonly used MNI normalization framework, and why native anatomical space might be better suited to discover subtle spatial WM tract alterations in individual patients. Nevertheless, MNI space remains a valuable standardized reference space for group comparisons. Besides, it is worth noting that our MNI space findings are specific only to the used standardized template, and that more sophisticated normalization methods including cohort-specific templates could potentially have yielded different results. Limitations of this study include its retrospective nature and relatively small sample size. In particular in the infiltration subgroup, the statistical power was limited due to its relatively small sample size (*n* = 10). Additionally, although based on expert consensus, the ground truth pattern classifications remained subjective, which may have introduced bias. Furthermore, previously mapped brain asymmetries could have influenced the MSP accuracy [37]. However, the MSP generation method described by Hu et al. (2003) was extensively validated for its robustness against asymmetries, volume rotations and noise [31]. Nevertheless, the assumption of a healthy contralateral tract could be a limitation of our mirroring-based analyses. While preserved geometry of infiltrated tracts could biologically reflect glioma integration into existing neural circuits, gliomas might also induce subtle structural remodeling in remote areas extending into the contralateral hemisphere, potentially compromising its role as a true control [38, 39]. Lastly, the current study did not evaluate to what extent the spatial metrics aligned with expert visual assessments, as no reliable predictive modeling was possible with our current dataset.

As the current findings exclusively concern the FAT, future research should validate these results in larger cohorts and other WM tracts that are commonly affected by gliomas, to increase their generalizability. This would also support future broader application of quantitative spatial metrics in tractography. It should be noted that our tract mirroring method in its current form might not be suitable for tracts with inherent structural asymmetries between hemispheres, such as the arcuate fasciculus [40]. Besides, a recent tractography study proposed a WM tract disruption category, supported by immunohistochemical staining, as part of a novel tumor-tract classification system [41]. Tissue-based validations of the WM tract alteration patterns could evaluate to what extent tractography captures each pattern in its current form, although such studies would be limited by the inability to biopsy functional WM tracts beyond tumor margins without risking neurological deficits. A potential application of spatial metrics such as the wDSC and HD lies in the field of radiomics, where quantitative imaging features are used to improve brain tumor diagnostics [42]. Therefore, we intend to explore the predictive value of the wDSC and HD to quantify visual displacement. Ultimately, research efforts aimed to distinguish between glioma subtypes, based on incorporating spatial metrics in advanced predictive classification models, may have future implications for surgical planning and patient counseling.

In conclusion, our findings highlight the potential of spatial metrics such as the wDSC and HD to objectively characterize FAT displacement in glioma. The current study provides the foundation for an objective, quantitative framework to evaluate spatial WM tract alterations in glioma. These results might advance non-invasive radiomics-based tumor subtype predictions.

## Supplementary Information

Below is the link to the electronic supplementary material.

Supplementary Material 1

## Data Availability

All data are stored in an institutional repository and available upon request.

## References

[1] Castellano A, Bello L, Michelozzi C, Gallucci M, Fava E, Iadanza A et al (2011) Role of diffusion tensor magnetic resonance tractography in predicting the extent of resection in glioma surgery. Neuro-Oncol 14:192–202 22015596 10.1093/neuonc/nor188 PMC3266379

[2] Henderson F, Abdullah KG, Verma R, Brem S (2020) Tractography and the connectome in neurosurgical treatment of gliomas: the premise, the progress, and the potential. Neurosurg Focus 48:E6 32006950 10.3171/2019.11.FOCUS19785 PMC7831974

[3] Sarubbo S, Vergani F, Yang JYM (2025) Tractography in brain tumor surgery: current clinical impact and future challenges. Brain Struct Funct 230:1–6 10.1007/s00429-025-02956-y 40493170

[4] Voets NL, Bartsch A, Plaha P (2017) Brain white matter fibre tracts: a review of functional neuro-oncological relevance. J Neurol Neurosurg Psychiatry 88:1017–1025 28710324 10.1136/jnnp-2017-316170

[5] Duffau H, Mandonnet E (2013) The onco-functional balance in surgery for diffuse low-grade glioma: integrating the extent of resection with quality of life. Acta Neurochir (Wien) 155:951–957 23447053 10.1007/s00701-013-1653-9

[6] Brennum J, Engelmann CM, Thomsen JA, Skjøth-Rasmussen J (2018) Glioma surgery with intraoperative mapping—balancing the onco-functional choice. Acta Neurochir (Wien) 160:1043–1050 29564654 10.1007/s00701-018-3521-0

[7] Mandonnet E, Duffau H (2018) An attempt to conceptualize the individual onco-functional balance: why a standardized treatment is an illusion for diffuse low-grade glioma patients. Crit Rev Oncol Hematol 122:83–91 29458793 10.1016/j.critrevonc.2017.12.008

[8] Laurent D, Freedman R, Cope L, Sacks P, Abbatematteo J, Kubilis P et al (2020) Impact of extent of resection on incidence of postoperative complications in patients with glioblastoma. Neurosurg 86:625–630 10.1093/neuros/nyz313 PMC7594111 31342060

[9] Sanai N, Berger MS (2008) Glioma extent of resection and its impact on patient outcome. Neurosurg 62:753–766 10.1227/01.neu.0000318159.21731.cf 18496181

[10] Stummer W, Reulen HJ, Meinel T, Pichlmeier U, Schumacher W, Tonn JC, ALA-Glioma Study Group (2008) Extent of resection and survival in glioblastoma multiforme: identification of and adjustment for bias. Neurosurg 62:564–576 10.1227/01.neu.0000317304.31579.17 18425006

[11] Duffau H (2022) White matter tracts and diffuse lower-grade gliomas: the pivotal role of myelin plasticity in the tumor pathogenesis, infiltration patterns, functional consequences and therapeutic management. Front Oncol 12:855587 35311104 10.3389/fonc.2022.855587 PMC8924360

[12] Giese A, Westphal M (1996) Glioma invasion in the central nervous system. Neurosurg 39:235–250 10.1097/00006123-199608000-00001 8832660

[13] Field AS, Alexander AL, Wu YC et al (2004) Diffusion tensor eigenvector directional color imaging patterns in the evaluation of cerebral white matter tracts altered by tumor. J Magn Reson Imaging 20:555–562 15390227 10.1002/jmri.20169

[14] Koga SF, Hodges WB, Adamyan H, Hayes T, Fecci PE, Tsvankin V et al (2024) Preoperative validation of edema-corrected tractography in neurosurgical practice: translating surgeon insights into novel software implementation. Front Neurol 14:1322815 38259649 10.3389/fneur.2023.1322815 PMC10801029

[15] Jellison BJ, Field AS, Medow J et al (2004) Diffusion tensor imaging of cerebral white matter: a pictorial review of physics, fiber tract anatomy, and tumor imaging patterns. AJNR Am J Neuroradiol 25:356–369 15037456 PMC8158568

[16] Witwer BP, Moftakhar R, Hasan KM et al (2002) Diffusion-tensor imaging of white matter tracts in patients with cerebral neoplasm. J Neurosurg 97:568–575 12296640 10.3171/jns.2002.97.3.0568

[17] Mahmoodi AL, Landers MJ, Rutten GJM, Brouwers HB (2023) Characterization and classification of spatial white matter tract alteration patterns in glioma patients using magnetic resonance tractography: a systematic review and meta-analysis. Cancers (Basel) 15:3631 37509291 10.3390/cancers15143631 PMC10377290

[18] Manan AA, Yahya NA, Taib NHM, Idris Z, Manan HA (2023) The assessment of white matter integrity alteration pattern in patients with brain tumor utilizing diffusion tensor imaging: a systematic review. Cancers (Basel) 15:3326 37444435 10.3390/cancers15133326 PMC10341056

[19] Gouttard S, Goodlett CB, Kubicki M, Gerig G (2012) Measures for validation of DTI tractography. Proc SPIE Int Soc Opt Eng 23:8314 10.1117/12.911546 PMC3864930 24353381

[20] Taha AA, Hanbury A (2015) Metrics for evaluating 3D medical image segmentation: analysis, selection, and tool. BMC Med Imaging 15:1–28 26263899 10.1186/s12880-015-0068-x PMC4533825

[21] Dice LR (1945) Measures of the amount of ecologic association between species. Ecology 26:297–302

[22] Sorensen T (1948) A method of establishing groups of equal amplitude in plant sociology based on similarity of species content and its application to analyses of the vegetation on Danish commons. Biol Skr 5:1–34

[23] Huttenlocher DP, Klanderman GA, Rucklidge WJ (1993) Comparing images using the HD. IEEE Trans Pattern Anal Mach Intell 15:850–863

[24] Dick AS, Garic D, Graziano P, Tremblay P (2019) The frontal aslant tract (FAT) and its role in speech, language and executive function. Cortex 111:148–163 30481666 10.1016/j.cortex.2018.10.015 PMC6461388

[25] Landers MJF, Brouwers HB, Kortman GJ, Boukrab I, De Baene W, Rutten GJM (2023) Oligodendrogliomas tend to infiltrate the frontal aslant tract, whereas astrocytomas tend to displace it. Neuroradiology 65:1127–1131 37127719 10.1007/s00234-023-03153-6 PMC10271893

[26] Meesters S, Landers M, Rutten GJ, Florack L (2023) Subject-specific automatic reconstruction of white matter tracts. J Digit Imaging 36:2648–2661 37537513 10.1007/s10278-023-00883-0 PMC10584769

[27] Tournier JD, Calamante F, Connelly A (2012) MRtrix: diffusion tractography in crossing fiber regions. Int J Imaging Syst Technol 22:53–66

[28] Mazziotta JC, Toga AW, Evans AC, Fox P, Lancaster J, Zilles K et al (2001) A probabilistic atlas and reference system for the human brain: International Consortium for Brain Mapping (ICBM). Philos Trans R Soc Lond B Biol Sci 356:1293–1322 11545704 10.1098/rstb.2001.0915 PMC1088516

[29] Tustison NJ, Cook PA, Holbrook AJ, Johnson HJ, Muschelli J, Devenyi GA et al (2021) The ANTsX ecosystem for quantitative biological and medical imaging. Sci Rep 11:9068 33907199 10.1038/s41598-021-87564-6 PMC8079440

[30] Van Essen DC, Smith SM, Barch DM, Behrens TE, Yacoub E, Ugurbil K, Wu-Minn HCP, Consortium (2013) The WU-Minn human connectome project: an overview. NeuroImage 80:62–79 23684880 10.1016/j.neuroimage.2013.05.041 PMC3724347

[31] Hu Q, Nowinski WL (2003) A rapid algorithm for robust and automatic extraction of the midsagittal plane of the human cerebrum from neuroimages based on local symmetry and outlier removal. NeuroImage 20:2153–2165 14683719 10.1016/j.neuroimage.2003.08.009

[32] Cousineau M, Jodoin PM, Garyfallidis E, Côté MA, Morency FC, Rozanski V et al (2017) A test-retest study on Parkinson’s PPMI dataset yields statistically significant white matter fascicles. Neuroimage Clin 16:222–233 28794981 10.1016/j.nicl.2017.07.020 PMC5547250

[33] Fedorov A, Beichel R, Kalpathy-Cramer J, Finet J, Fillion-Robin JC, Pujol S et al (2012) 3D Slicer as an image computing platform for the Quantitative Imaging Network. Magn Reson Imaging 30:1323–1341 22770690 10.1016/j.mri.2012.05.001 PMC3466397

[34] IBM Corp (2023) IBM SPSS Statistics for Windows, Version 29.0.1.0. Armonk. IBM Corp, NY

[35] Greene C, Cieslak M, Grafton ST (2018) Effect of different spatial normalization approaches on tractography and structural brain networks. Netw Neurosci 2:362–380 30294704 10.1162/netn_a_00035 PMC6145854

[36] Kim J, Avants B, Patel S, Whyte J, Coslett BH, Pluta J et al (2008) Structural consequences of diffuse traumatic brain injury: a large deformation tensor-based morphometry study. NeuroImage 39:1014–1026 17999940 10.1016/j.neuroimage.2007.10.005 PMC2323832

[37] Toga AW, Thompson PM (2003) Mapping brain asymmetry. Nat Rev Neurosci 4:37–48 12511860 10.1038/nrn1009

[38] McAlpine H, Rosier M, Rozario J, Wang X, Wimmer VC, Guzulaitis R et al (2025) Increased neural excitability and glioma synaptic activity drives glioma proliferation in human cortex. Nat Neurosci 29:350–357 41298888 10.1038/s41593-025-02149-0

[39] Wei Y, Li C, Cui Z, Mayrand RC, Zou J, Wong ALKC et al (2023) Structural connectome quantifies tumour invasion and predicts survival in glioblastoma patients. Brain 146:1714–1727 36189936 10.1093/brain/awac360 PMC10115235

[40] Vernooij MW, Smits M, Wielopolski PA, Houston GC, Krestin GP, van der Lugt A (2007) Fiber density asymmetry of the arcuate fasciculus in relation to functional hemispheric language lateralization in both right- and left-handed healthy subjects: a combined fMRI and DTI study. NeuroImage 35:1064–1076 17320414 10.1016/j.neuroimage.2006.12.041

[41] Hu J, Bao H, Liu X, Fang S, Yan Z, Wang Z et al (2025) Glioma-white matter tract interactions: a diffusion magnetic resonance imaging-based 3-tier classification and its clinical relevance. Neuro-Oncol 27:1888–1898 39946091 10.1093/neuonc/noaf036 PMC12417821

[42] Yi Z, Long L, Zeng Y, Liu Z (2021) Current advances and challenges in radiomics of brain tumors. Front Oncol 11:732196 34722274 10.3389/fonc.2021.732196 PMC8551958

