# Supplementary material for: Objective characterization of displacement of the frontal aslant tract in low-grade glioma: a quantitative tractography study
Source: Neuroradiology. 2026 Apr 1;68(7):1993–2003. doi: 10.1007/s00234-026-03968-z (PMC13407475; doi:10.1007/s00234-026-03968-z)
Supplement: Supplementary file 1 — Supplementary Material 1 [file 234_2026_3968_MOESM1_ESM.docx]

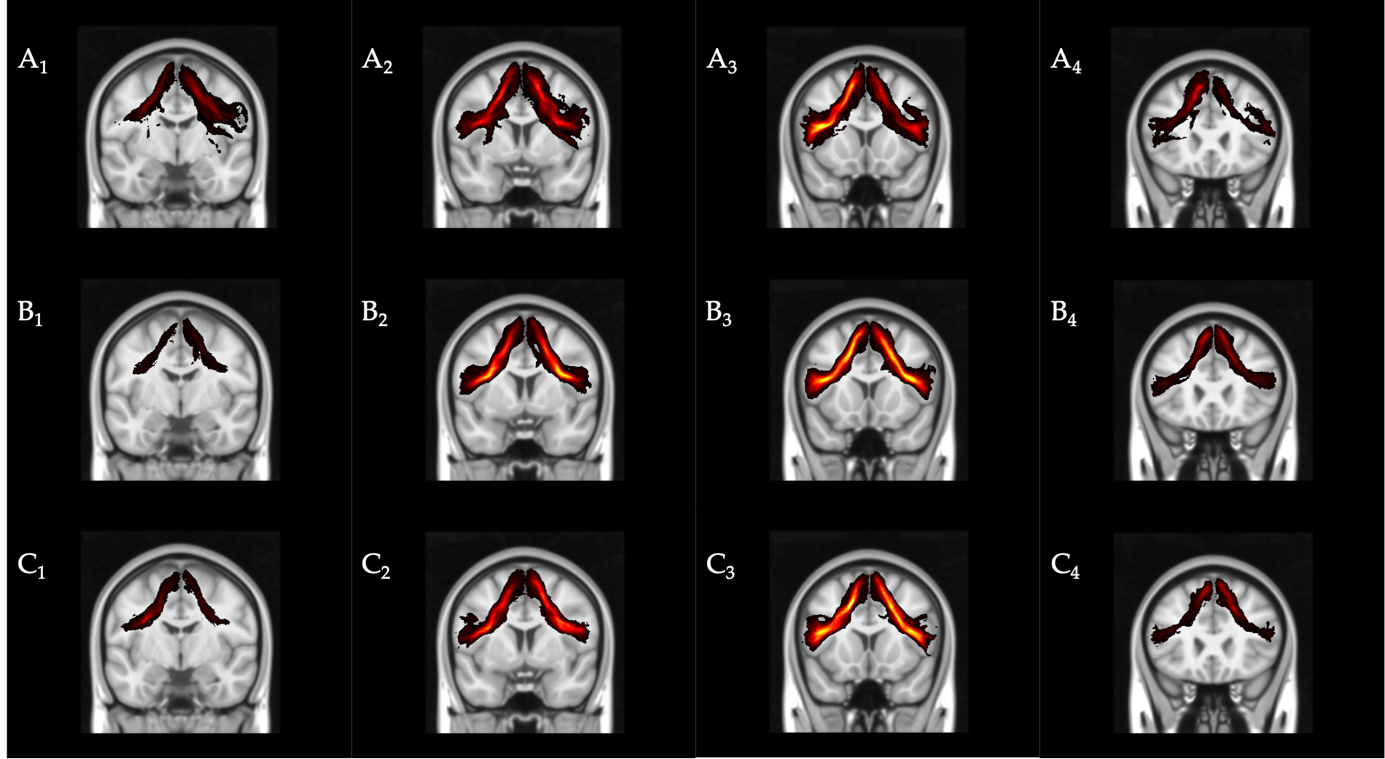


**Supplementary Fig. 1 Probabilistic heatmaps of MNI normalized patient streamlines**

Coronal slices of the MNI-152 template show the probability on a scale from 0 (black) to 1 (yellow) of streamlines passing a certain voxel, for each white matter tract alteration pattern.

**A_1-4_)** Combined displacement and infiltration group. **B_1-4_)** Displacement group **C_1-4_)** Infiltration group.
